# Case Report: Can we differentiate mesothelioma from inflammatory pericardial constriction preoperatively?

**DOI:** 10.12688/f1000research.23998.1

**Published:** 2020-06-04

**Authors:** Syed Shahmeer Raza, Irfan Ullah, Saira Kainat Awan, Muhammad Daniyal Nadeem, Gulsam Bashir

**Affiliations:** 1 Khyber Medical College, Peshawar, KP, 25000, Pakistan; 2 Gastroentrology, Prince Charles Hospital, Merthyr Tydfil, Merthyr Tydfil, UK; 3 Kabir Medical College, Peshawar, Pakistan

**Keywords:** Mesothelioma, Cardiac Surgery, Pericarditis

## Abstract

Primary malignant pericardial mesothelioma (PMPM) is a rare cardiac tumor. The patient usually presents with pericardial constriction, usually misdiagnosed and wrongly managed. We present the case of a 21-year-old woman with a history of pericarditis and cardiac tamponade. The patient was referred from a clinic due to nausea, vomiting and ascites with lower extremity edema, soft and watery diarrhea, and right upper quadrant pain. Surgery (sternotomy and partial pericardiectomy) was proposed after looking at the different relevant investigations; it was not until that the patient was operated on that it was established that this wasn't a mere constriction but a malignancy. The patient shortly died after the operation. Pathology made a diagnosis of PMPM. Along with the classical symptoms those who present with level 1 thoracic adenopathy a decision to operate should be very carefully made, this may lead to a misdiagnosis of PMPM which postoperatively results in patient's death.

## Introduction

The overall outcome of primary malignant pericardial mesothelioma (PMPM) is very poor. Primary pericardial tumors are very rare, 40 times less common than metastatic ones. The patient usually presents with a pericardial constriction and is consequently misdiagnosed and wrongly managed, which can lead to death either on the operating table or right after the surgery ^1– 2^. So far there has not been any curative treatment proposed for PMPM. Palliative management is the mainstay of treatment for such tumours, which mostly includes chemotherapy and pericardiectomy. However, even with these measures, the patient doesn't survive for long ^3^.

## Case report

A 21-year-old woman with a past medical history of pericarditis and cardiac tamponade presented to the Emergency Department of our hospital after a referral from a clinic due to nausea, vomiting and ascites with lower extremity oedema, soft and watery diarrhoea, right upper quadrant pain, scleral icterus, abdominal distention, oedema, rash and joint pain. 

At two months prior to presentation she developed pleuritic chest pain and arthralgias and was diagnosed with cardiac tamponade. Pericardiocentesis was performed, which was negative for any malignant cells, showing only mild nonspecific inflammation. At that time, she had a negative workup for HIV, hepatitis, antinuclear antibody and Anti-Smith antibodies. She was hospitalised for 10 days and discharged without medication.

Upon her arrival to the Emergency Room, she complained of not being able to keep food down and complained of a distended abdomen when taking meals, though she could tolerate some fluid. The distention improved with emesis. She reported a weight loss of 20 lb (approximately 9 kg) in the past month. She was not taking any medication at presentation. The Cardiology Department presented the case to our service for Surgical consultation in the Cardiothoracic Surgery Department of our Hospital.

Systemic review showed the patient had fatigue, shortness of breath, cough, weight change and arthralgias. Upon physical examination, she had a rash in the lower extremities and back. Heart exam showed that the point of maximal impact was not displaced. No murmurs, gallop or bruit or raised jugular venous pressure. Bilaterally edema was positive in both her legs

The ECG showed low voltage QRS, sinus tachycardia and T wave abnormality. Chest X ray Impression- the cardiomediastinal silhouette Borderline enlarged. Cardiac catheterization-final anatomic diagnosis: constrictive pericarditis (by echo and cardiac magnetic resonance imaging (MRI)). The recommendation was to evaluate for pericardial stripping. 

**Figure 1.**
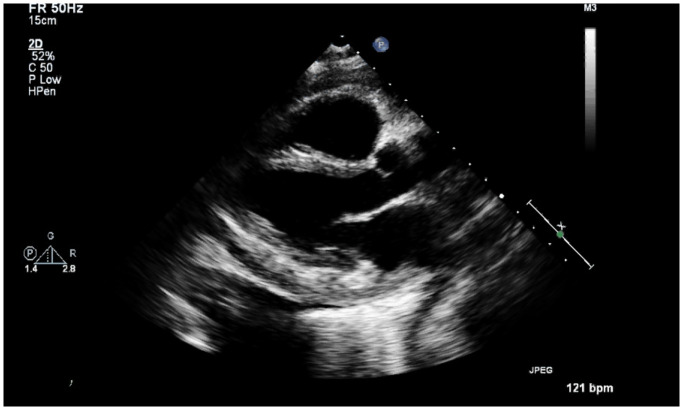
Echocardiography shows markedly thickened pericardium and abnormal soft tissue echoes anterior mediastinum.

Echocardiography showed that left ventricular systolic function was moderately decreased. Ejection Fraction was 30 (± 5). The right ventricular systolic function was moderate to severely decreased, along with abnormal soft tissue echoes in the anterior mediastinum. Constrictive Pericarditis with markedly thickened Pericardium was appreciated ( Figure 1).

**Figure 2.**
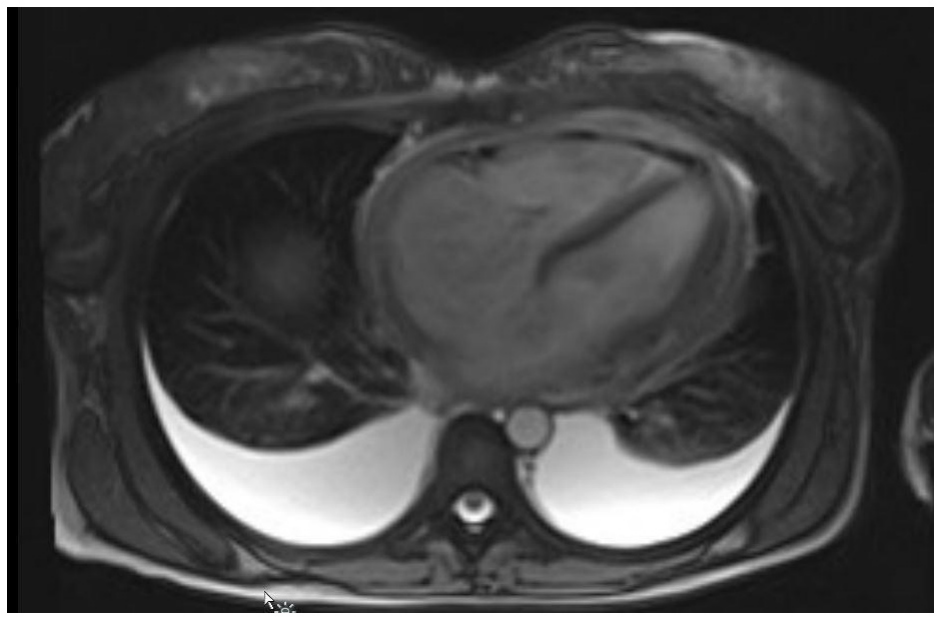
Magnetic resonance imaging shows diffuse pericardial thickening with only minimal amount of effusion.

**Figure 3.**
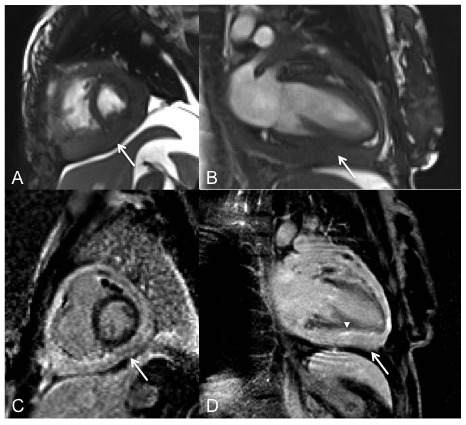
Cardiac magnetic resonance with intravenous contrast. Short axis ( **A**) and 2 chamber ( **B**) images of steady-state free precession sequence demonstrating diffuse pericardial thickening with loss of normal interphase between the myocardium and pericardium (arrow). Short axis ( **C**) and two-chamber ( **D**) views of inversion recovery images obtained approximately 10 minutes after IV injection of gadolinium, showing diffuse pathological pericardial enhancement (arrows) and to a lesser extent epicardial enhancement and loss of normal myocardial - epicardial interphase (arrowhead).

MRI was conducted to further examine, as echocardiography alone was difficult to interpret making it hard to arrive at a diagnosis. Cardiac MRI showed diffuse pericardial thickening with only minimal amount of effusion. The chest wall appeared normal ( Figure 2). Constrictive pericarditis was the main concern and cardiac magnetic resonance (CMR) was performed for further characterization. The diagnosis was constrictive pericarditis with markedly thickened pericardium ( Figure 3).

**Figure 4.**
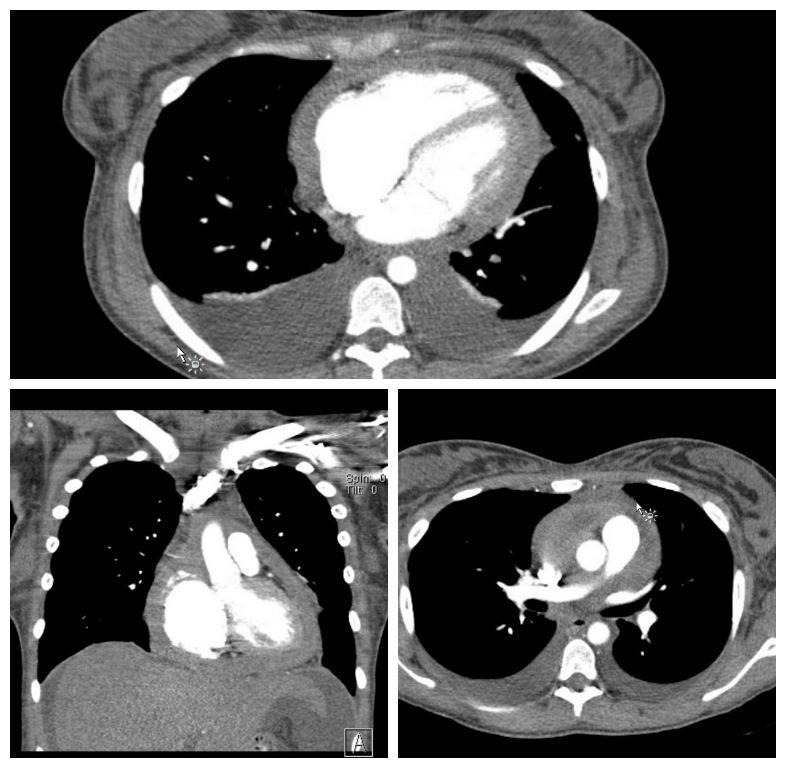
The different views and sections of a computed tomography scan showing a markedly thickened pericardium and level 1 thoracic adenopathy.

Computed tomography findings showed there was an underlying Constrictive Pericarditis with a markedly thickened Pericardium. Also observed were anterior mediastinal lymphadenopathy, moderate pleural effusion and associated atelectasis ( Figure 4).

The patient had a full cardiology work-up on the day of presentation, including echocradiogram, cardiac catheterisation and MRI. We agreed that pericardiectomy would be warranted given her degree of constriction. After extensive consideration and discussion with the patient and her family she agreed to undergo sternotomy and pericardiotomy. She was taken to the operating room two days after presenting and after opening the chest, the pericardium was noted to be thick and firm. When we began to dissect the pericardium off the right ventricle, it seemed it was invading the right ventricular mass. We therefore began to suspect a malignant tumor, and after careful stripping of the densely adherent pericardium, a tissue sample was sent to pathology. A frozen section revealed findings consistent with a malignant tumor, possibly a mesothelioma or metastatic breast cancer. Partial pericardiectomy was performed as planned. Shortly after coming off bypass it was clear that the patient was not tolerating cardiac function independently, and high-dose norepinephrine (0.14 µg/kg/min IV infusion), epinephrine (1 mg bolus) and vasopressin (40 Units IV push after second epinephrine dose) was administered. She shortly went into ventricular fibrillation arrest. Multiple shocks and open cardiac massage achieved a viable rhythm with inotropic and intra-aortic balloon pump (IABP) assistance. The chest was left open and she was transferred to the Surgical Intensive Care Unit (SICU) in critical condition. Shortly after arriving the SICU from the operating room she became hypotensive. Multiple efforts including multiple shocks, high dose epinephrine and continuous CPR were made to resuscitate her. After half an hour it was evident that we were unable to resuscitate her. The patient then developed electromechanical dissociation and died shortly after.

Final pathologic diagnosis of the specimen taken as excisional biopsy revealed biphasic malignant mesothelioma alongside signs of level 1 thoracic adenopathy, which is consistent with cardiac neoplasia PMPM. The morphologic features of the specimen and the immunophenotypic findings support diagnosis of pericardial malignant mesothelioma. Retrospective evaluation of the CMR revealed subtle loss of the normal interface between the epicardial myocardium and the pericardium on steady-state free precession and inversion recovery delayed enhancement images which suggested myocardial invasion by the pericardial process. This should raise concern for neoplastic etiology ( Figure 4).

## Discussion

As discussed earlier, this disease is rare and is difficult to diagnose and manage. This is not only tiresome and challenging but can be misleading requiring multiple kinds of imaging techniques and approaches ^1, 2^. The overall outcome after all medical or surgical intervention is also relatively poor. The patient is misdiagnosed for pericardial constriction which leads to unnecessary surgical intervention and ultimately results in the death of the patient. Asbestos has always been thought to be the causative agent and is linked with it but not such evidence has yet been documented in the medical literature. A large number of pericardial tumors are of secondary or metastatic nature, with few of primary nature, although the etiology of such tumors is still not known ^3– 5^. The disease mimics other diseases of cardiac origin like cardiac tamponade, coronary cardiac diseases and constrictive pericarditis, which further adds to the misery of misdiagnosis. PMPM has a very low incidence—nearly one in 40 million. Notably, a diagnosis of PMPM is mostly made post-mortem and rarely ante-mortem. Palliative management is the mainstay of treatment for such tumors, mostly including chemotherapy and pericardiectomy, but even with this, the patient is unlikely to survive for long.

PMPM is characterized by a sudden onset of symptoms, with patient history mostly misleading and unclear. The tumor may occasionally spread to surrounding mediastinal structures and cause manifestations, but it usually mimics other diseases like myxomas or a tubercular pericarditis. Emboli, conduction blocks and distant metastasis have also been reported ^6^.

To our knowledge, no information is available about the accuracy of cytologic diagnosis of pericardial mesothelioma; however, in a large study of cytologic samples in 517 cases of pleural mesothelioma, definitive diagnosis was only made in 73% of cases, with a further 13% being suspicious ^7^. Only 25% of reported cases of pericardial mesothelioma were diagnosed antemortem ^5^. Though surgery is often discussed, in general outcomes are poor and not significantly impacted by surgical intervention ^8^.

A key question for surgeons is if there are findings suggestive of mesothelioma that may help avoid unsuccessful procedures. In the presence of very thickened pericardium, CMR might help differentiate the process preoperatively. Ohnishi *et al.* reported that gadolinium DTPA-enhanced MRI led the authors to suspect a tumor though in their patient there was a discrete soft tissue mass (5). In our case, the mesothelioma was diffuse, not localized, though the loss of the epicardial-myocardial interface was suggestive of tumor invasion. Had mesothelioma been suspected prospectively, the procedure would have been a biopsy, rather than attempted pericardiectomy.

A varied and multidimensional approach needs to be considered by the physician in managing patients who present with such symptoms. The investigations need to be thoroughly evaluated and a decision should then be made. Along with the classical symptoms those who present with level 1 thoracic adenopathy, a decision to operate should be very carefully made in such patients, this may lead to a misdiagnosis of PMPM which may postoperatively result in patient's death. Level 1 Adenopathy presenting with Classical S/S and Pericardial constriction should by far be considered diagnostic for PMPM.

## Data availability

All data underlying the results are available as part of the article and no additional source data are required.

## Consent

Written informed consent for publication of their clinical details and clinical images was obtained from the parents of the patient.
