# Supplementary material for: Case Report: Can we differentiate mesothelioma from inflammatory pericardial constriction preoperatively?
Source: F1000Res. 2020 Jun 4;9:513. [Version 1] doi: 10.12688/f1000research.23998.1 (PMC8825643; doi:10.12688/f1000research.23998.1)
Supplement: Peer review history

# Peer review: Reviewer response for version 1

**DOI:** 10.5256/f1000research.26474.r99934

**Published:** 2022-02-08

**Authors:** Mehmet Ali Bedirhan

**Affiliations:** 1 Department of Thoracic Surgery, Yedikule Chest Diseases and Chest Surgery Training and Research Hospital, İstanbul, Turkey

Firstly, I am sorry for the loss of patients. Primary pericardial mesothelioma (PPM) is a rare disease with unfortunate poor prognosis. Most of the PPM patients (75%) receive diagnosis in late stage disease forms (McGehee *et al.* (2019 ^1^)). PPM is not a common tumor which makes its diagnosis cumbersome. Clinical dompt and experience is mandatory for its diagnosis; all in all who thinks a 21-year old can have PPM. Nevertheless, my first PPM case was 12-year old male in 1986 who have managed to live only a few months.

 Most pericardial tumors are secondary lesions due to local spread of lung and mediastinal tumors or metastatic lesions originating from lung cancer, breast cancer, lymphoma or melanoma. Primary malignant tumors are rare and 50% are mesotheliomas. Other causes are sarcoma, hemangioma and teratoma. 

 In differential diagnosis, radiological examinations such as computed tomography may not help differentiate PPM from inflammatory constrictive pericarditis (ICP). In both diseases, clinician may observe effusion, cardiomegaly, ascite or pedal edema. Cardiac MR is recommended when tumoral invasion of the myocardium exist (Cosyns *et al.* (2015 ^2^)). CMR does not provide a histological diagnosis; but provides clues for diagnosis and tissue characterization, with excellent soft tissue separation and wide field of view, and may allow the origin, extent and borders of the tumor to be determined. Most tumors will retain contrast and show delayed contrast (wash-out sign) (Wang *et al.* (2003 ^3^) and Hoffmann *et al*. (1998 ^4^)) .   

 As an invasive diagnostic method of ICP, right sided cardiac catheterization is pretty essential to reach a precise diagnosis. During this method, elevated right atrial pressure, plateau sign or no pressure change during cardiac cycles may be the findings. In addition, ventricular ejection fraction is preserved in ICP whereas it is affected in PPM (Cosyns *et al.* (2015 ^2^)).

 Lastly, dissection of constrictive layer start from left side of the heart (reverse direction of intracardiac blood flow). Otherwise pulmonary edema develops and acute mortal right sided failure may occur. 

 PPM remains a rare and poorly understood malignancy of unclear etiology (McGehee *et al.* (2019 ^1^)). Despite these diagnostic tools and experience we sometimes still in trouble. I personally thank the authors who share their practice with us.

Are enough details provided of any physical examination and diagnostic tests, treatment given and outcomes?

Yes

Is the case presented with sufficient detail to be useful for other practitioners?

Yes

Is sufficient discussion included of the importance of the findings and their relevance to future understanding of disease processes, diagnosis or treatment?

Yes

Is the background of the case’s history and progression described in sufficient detail?

Yes

Reviewer Expertise:

Thoracic & cardiovascular surgery

I confirm that I have read this submission and believe that I have an appropriate level of expertise to confirm that it is of an acceptable scientific standard.

## References

[1] : Treatment and Outcomes of Primary Pericardial Mesothelioma: A Contemporary Review of 103 Published Cases. *Clin Lung Cancer* .20(2) : 10.1016/j.cllc.2018.11.008 e152-e157 10.1016/j.cllc.2018.11.008 30594459

[2] : European Association of Cardiovascular Imaging (EACVI) position paper: Multimodality imaging in pericardial disease. *Eur Heart J Cardiovasc Imaging* .2015;16(1) : 10.1093/ehjci/jeu128 12-31 10.1093/ehjci/jeu128 25248336

[3] : CT and MR imaging of pericardial disease. *Radiographics* .2003;23 Spec No: 10.1148/rg.23si035504 S167-80 10.1148/rg.23si035504 14557510

[4] : Cardiac and paracardiac masses. Current opinion on diagnostic evaluation by magnetic resonance imaging. *Eur Heart J* .1998;19(4) : 10.1053/euhj.1997.0788 553-63 10.1053/euhj.1997.0788 9597403

# Peer review: Reviewer response for version 1

**DOI:** 10.5256/f1000research.26474.r64883

**Published:** 2020-06-17

**Authors:** Muhammad Aasim

**Affiliations:** 1 Hayatabad Medical Complex, Peshawar, Pakistan

This Case Report is very much pertinent to the practice of cardiothoracic surgeons treating constrictive pericarditis. Primary Malignant Pericardial Mesothelioma is a rare cause of constrictive pericarditis, but associated with poor surgical outcome, hence it shall be understood and diagnosed well. This case is adequately described and presented appropriately to guide the diagnosing cardiologists and treating cardiothoracic surgeons.

Are enough details provided of any physical examination and diagnostic tests, treatment given and outcomes?

Yes

Is the case presented with sufficient detail to be useful for other practitioners?

Yes

Is sufficient discussion included of the importance of the findings and their relevance to future understanding of disease processes, diagnosis or treatment?

Yes

Is the background of the case’s history and progression described in sufficient detail?

Yes

Reviewer Expertise:

Cardiac Surgery, Adult Cardiac Surgery, Congenital Cardiac Surgery, Cardiothoracic Surgery, Cardiovascular Surgery.

I confirm that I have read this submission and believe that I have an appropriate level of expertise to confirm that it is of an acceptable scientific standard.
